# Meffil: efficient normalization and analysis of very large DNA methylation datasets

**DOI:** 10.1093/bioinformatics/bty476

**Published:** 2018-06-21

**Authors:** J L Min, G Hemani, G Davey Smith, C Relton, M Suderman

**Affiliations:** 1 MRC Integrative Epidemiology Unit, University of Bristol, Bristol, UK; 2 Bristol Medical School, University of Bristol, Bristol, UK

## Abstract

**Motivation:**

DNA methylation datasets are growing ever larger both in sample size and genome coverage. Novel computational solutions are required to efficiently handle these data.

**Results:**

We have developed *meffil*, an R package designed for efficient quality control, normalization and epigenome-wide association studies of large samples of Illumina Methylation BeadChip microarrays. A complete re-implementation of functional normalization minimizes computational memory without increasing running time. Incorporating fixed and random effects within functional normalization, and automated estimation of functional normalization parameters reduces technical variation in DNA methylation levels, thus reducing false positive rates and improving power. Support for normalization of datasets distributed across physically different locations without needing to share biologically-based individual-level data means that *meffil* can be used to reduce heterogeneity in meta-analyses of epigenome-wide association studies.

**Availability and implementation:**

https://github.com/perishky/meffil/

**Supplementary information:**

Supplementary data are available at *Bioinformatics* online.

## 1 Introduction

DNA methylation is the addition of methyl groups to cytosine bases in the DNA sequence, most often in the context of a CpG dinucleotide, a cytosine followed by a guanine. The addition or loss of methyl groups is often associated with changes in gene expression, and through epigenome wide associations studies (EWAS) it has been shown to associate with a wide range of complex traits. A number of technologies have been developed for interrogating DNA methylation including microarrays and sequencing-based methods. The Illumina Infinium HumanMethylation450 BeadChip (450k array) can be used to measure DNA methylation of 485k CpG sites, comprising just under 2% of the total genomic CpG content mainly clustered around the transcription start sites (Michels *et al.*, 2013). The new Illumina Infinium MethylationEPIC BeadChip (EPIC array) expands this coverage to ∼850k sites to include enhancer regions identified by ENCODE (Hong *et al.*, 2016) and FANTOM5 (Andersson *et al.*, 2014).

Batch effects present a well-known challenge to microarray analysis (Teschendorff *et al.*, 2011), particularly in datasets composed of thousands of samples since they cannot all possibly be processed at the same times and by the same technical personnel (Leek *et al.*, 2010). This unwanted variation can increase both false negative and false positive rates if correlated with the outcome of interest, and controlling for this is not trivial, especially as sample sizes continue to grow.

Following the popularity of quantile normalization for analyzing gene expression microarrays (Bolstad *et al.*, 2003), many variations based on quantile normalization have been developed for DNA methylation microarrays (Lehne *et al.*, 2015; Teschendorff *et al.*, 2013; Touleimat and Tost, 2012), however, all assume that global methylation does not vary between samples (Hicks and Irizarry, 2015). When this does not hold, most notably between tumor and normal samples, between different tissue types, or when there are batch differences between cases and controls, quantile normalization can remove biological variation along with technical variation [e.g. (Fortin *et al.*, 2014b; Heiss and Brenner, 2015)]. A feature of 450k and EPIC arrays is the inclusion of control probes–probes that do not assay biological variation and only vary due to technical effects. Functional normalization (FN; Fortin *et al.*, 2014b) exploits control probes to separate biological variation from technical variation, and its performance compares favorably to other approaches (Fortin *et al.*, 2014b; Heiss and Brenner, 2015; Lehne *et al.*, 2015; Liu and Siegmund, 2016; Maksimovic *et al.*, 2015).

Many DNA methylation datasets using 450k and EPIC arrays have now been generated independently and are being used in EWAS to discover associations between CpG sites and a variety of exposures, complex traits and disease risks. Much like genome-wide association studies (GWAS), the widespread use of the Illumina platform has made possible large-scale meta-analyses organized by international consortium-based efforts that are combining ever larger numbers of subjects to reach statistical power to detect robust associations. However, unlike GWAS, it comes with a number of crucial challenges that have not been fully resolved. First, most existing software tools for quality control and normalizing DNA methylation levels were not designed to handle datasets comprising thousands of samples, and recently developed tools in R (Gorrie-Stone, 2018) and Java (Almeida *et al.*, 2017) may not provide desired functionality. Second, sharing of individual-level data is prohibited due to ethical considerations, so meta-analyses are liable to encounter heterogeneity introduced when datasets are normalized independently. Third, there is currently no universally accepted standard approach that addresses all aspects of dataset quality control and normalization of large datasets. Fourth, there is no standardized approach for selecting and comparing statistical models for EWAS, including selection and generation of covariates. Fifth, there is currently no standard for sharing quality control information and EWAS findings.

**Fig. 1.**
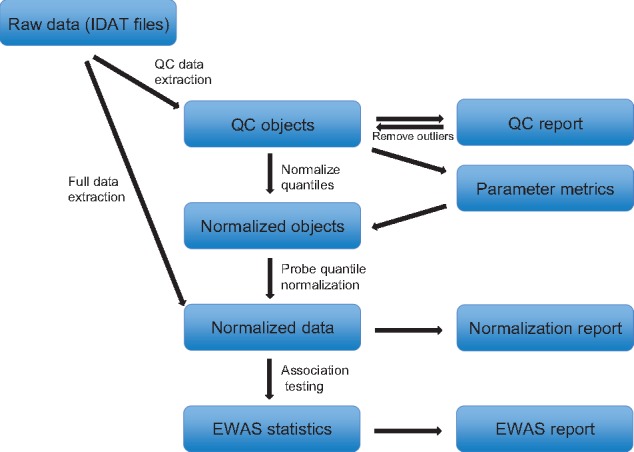
The workflow of *meffil*

We have developed *meffil* (Efficient algorithms for analyzing DNA methylation data) to provide solutions in a user-friendly and open source R package (https://github.com/perishky/meffil). Figure 1 shows the *meffil* work-flow from raw data to quality control to normalized data to EWAS. Meffil includes functionality for identifying low quality methylation measurements, discovering and rectifying sample mismatches, merging datasets containing both 450k and EPIC arrays, removing confounding effects of cell type heterogeneity and assessing the quality of observed associations. In this paper, we describe its implementation and evaluate the computational and statistical advantages that it achieves, while demonstrating where limitations might still exist.

## 2 Materials and methods

### 2.1 Data

Full details of the Accessible Resource for Integrated Epigenomic Studies (ARIES; Relton *et al.*, 2015) and Genetics of Overweight Young Adults (GOYA; Paternoster *et al.*, 2011) datasets are provided in the Supplementary Material. DNA methylation was quantified in bisulfite-converted genomic DNA using 450k arrays for all samples. Some samples were removed due to genotype and gender mismatches and methylation quality (low detection scores, low number of beads, methylated/unmethylated ratio, strong dye-bias, post-normalization checks). Samples were normalized using FN using *meffil.* After normalization we checked for batch effects including bisulfite-conversion plate (‘plate’) and beadchip (‘slide’).

### 2.2 Implementation of *meffil*

*Meffil* is designed around a re-implementation of FN as implemented in the *minfi* R package (Fortin *et al.*, 2014b). Output using default settings and without enhancements is therefore identical to *minfi* (Aryee *et al.*, 2014)*. Meffil* uses the *illuminaio* R package (Smith *et al.*, 2013) to parse Illumina IDAT files into QC objects (Fig. 1) which contain raw control probe summaries, quantile distributions of raw probe intensities, poor quality probes based on detection *P*-values and number of beads, predicted sex (Aryee *et al.*, 2014), predicted cellular composition (Houseman *et al.*, 2012) when a cell type reference is specified and batch variable values. As in minfi, probe intensities are dye-bias and background corrected using the ‘noob’ method (Triche *et al.*, 2013). Control probes are summarized as 42 different control types in a control matrix with one row for each control type and one column for each sample.

This summary object is all that is needed to perform quality control, sample and CpG site filtering, identification of batch effects and the normalization of sample quantiles, the first normalization step of FN. In this step, probe intensity quantiles are normalized between samples by fitting linear models with these quantiles to the top principal components of the control matrix. The resulting quantile residuals for each QC object are retained as a set of normalized quantiles which are then used in the second normalization step where the raw probe intensities for each sample are adjusted to conform to its set of normalized quantiles.

This memory-reducing innovation makes it possible to perform the second normalization step on small subsets of the dataset, each at different times or on different compute servers. Parallelization of the normalization is possible when either a single compute server has multiple processors or the normalization is being performed on a compute cluster. After the second normalization step has been completed for each individual sample, the resulting normalized methylation data subsets may be merged into a single dataset for DNA methylation analyses. The order of or server on which the samples were normalized does not affect the final normalized values in any way.

### 2.3 Quality control features

In *meffil*, quality control reports can be generated in order to uncover variation due to technical artefacts, identify outliers and flag poor quality probes and samples using detection *P*-values, number of beads, ratio of unmethylated/methylated signal, dyebias and control probe checks. The report also provides checks for sample swap detection using SNP discordance between methylation and genotype arrays as well as a gender check (Supplementary Fig. S1).

*Meffil* generates a normalization report with coefficients plots comparing the strength of associations between batch variables with control probes and with normalized data. The report contains a table with ANOVA F and post-hoc *t*-statistics that pass a user-defined significance threshold to identify problematic batches, e.g. a specific slide with technical artefacts that are not sufficiently resolved by normalization (Supplementary Fig. S2). All reports are generated in markdown and HTML.

### 2.4 Extending FN to reduce technical variation

*Meffil* provides two new features to reduce technical variation: (i) a method to identify the number of principal components that minimizes the residual variance unexplained by the given number of principal components. Residual variance is calculated under a 10-fold cross-validation scheme in order to avoid overfitting. (ii) We observed that FN failed to completely remove the variance due to certain technical artefacts such as sample slide or slide row. To address this, we allow the user to normalize sample quantiles using additional fixed and random effects. Random effects are handled using the *lme4* R package (Bates *et al.*, 2015).

### 2.5 EWAS pipeline

To deliver a comprehensive and integrated toolkit for methylation analysis, *meffil* also provides an EWAS pipeline. Linear regression models are fitted using *limma* (Ritchie *et al.*, 2015). Confounding effects are handled by including appropriate covariates in the EWAS. By default, *meffil* fits four different regression models: no covariates, only supplied covariates, supplied and surrogate variables obtained by surrogate variable analysis (SVA; Leek *et al.*, 2012; Leek and Storey, 2007) and supplied and surrogate variables obtained by independent surrogate variable analysis (ISVA; Teschendorff *et al.*, 2011). *Meffil* allows estimation of cellular composition using the Houseman algorithm (Houseman *et al.*, 2012) from DNA methylation profiles based on several publicly available blood reference datasets including three cord blood references (Bakulski *et al.*, 2016; de Goede *et al.*, 2015; Gervin *et al.*, 2016) and one peripheral blood reference (Reinius *et al.*, 2012) or from user-supplied references.

EWAS results are summarized in a report that includes quantile-quantile, Manhattan, covariate and variable-of-interest plots as well as tables and scatterplots showing the strongest as well as user-defined candidate CpG site associations. Outputs are displayed to allow comparison between each of the different EWAS models (Supplementary Fig. S3).

### 2.6 Analysis that protects study participant privacy

Because the control probes capture only technical variation, they are fundamentally non-disclosive. It is possible to use *meffil* to normalize datasets residing on distinct servers together while sharing only the control probe summaries and probe intensity quantiles between the two servers. This information cannot be used to identify individuals and should not violate most cohort participant privacy agreements. Actual phenotype or DNA methylation levels need never be shared.

## 3 Results

### 3.1 Automated normalization for heterogeneous data with improved computational efficiency

#### *3.1.1* Computational efficiency

Our original motivation for creating *meffil* was an inability to successfully normalize ∼5400 450k arrays using available software tools and computational resources. The main impediment was the large memory requirement of loading all data into memory before normalization could be initiated. We discovered, however, that FN (Fortin *et al.*, 2014b) could be reimplemented in a way that uses a small fraction (∼1/20) of the memory required by the entire dataset. In particular, we realized that FN could be completed one sample at a time while holding in memory a relatively small summary of probe intensities for each sample. The summary consists of a control probe matrix and probe intensity quantiles. After the summary has been collected, FN then proceeds to normalize intensity quantiles by removing control probe variation. Normalized methylation levels for each sample can then be derived from the normalized quantiles independently of all other samples.

To minimize running time, the *meffil* implementation makes use of the R parallel package (R Core Team, 2014) to allow normalization of multiple samples simultaneously. Normalization of 5469 450k arrays took 3 h on a compute server with 64 Gb of RAM and 16 processors. A comparison shows that the memory requirements to normalize the same dataset using another popular software tool, minfi, were much larger (Table 1). Most other popular packages (Assenov *et al.*, 2014; Morris *et al.*, 2014) that provide FN capability are simply wrappers for the minfi implementation. Two recently developed tools, bigMelon (Gorrie-Stone, 2018) and DiMmeR (Almeida *et al.*, 2017) were also specifically designed to normalize large datasets. Although neither implements FN, we provide their performance characteristics for comparison.

**Table 1.** Comparison between software packages on a server with 16 available processors

	meffil	minfi	meffil	bigMelon^a^	bigMelon^b^	diMmeR
Number of samples	1000	1000	5469	5469	5469	5469
Normalization method	FN	FN	FN	Dasen	Dasen	QN
Platform	R	R	R	R	R	Java
Size of summary (Gb)^c^	0.2		0.8			
Memory (Gb)	3/5^d^	15	3/67^d^	57	12	4.4
Time (min)	16	54	180	350	450	82
Size of output (Gb)^e^	3.5	2.8	17	90	90	

a bigMelon applied with chunksize set to 500.

b bigMelon applied with chunksize set to 100.

c Only meffil generates a summary object.

d If the output from meffil is a matrix in R, then memory use peaks at 67 Gb. If the output is saved to ‘gdsfmt’(Zheng *et al.*, 2012, 2017) file like bigMelon, then the memory use peaks at 3 Gb. We note that the running time will be the same for both options.

e DimMeR does not save output until after a permutation-based EWAS is run. We terminated analysis after normalization so output size was not determined.

#### *3.1.2* Scalable pipeline and reporting mechanisms

Normalization and analysis of datasets, particularly large datasets, is rarely automatic and requires interactive problem-solving. Ideally, then, analysis tools should reflect this, allowing for some level of automation while also allowing high-level tasks to be broken down into more specific tasks with customizable solutions. Graphical user interface packages, which are most convenient for users, are often not available on computational servers or high-performance computing cluster. Graphical interfaces like shinyMethyl (Fortin *et al.*, 2014a) and MethylAid (van Iterson *et al.*, 2014) handle this problem by extracting data summaries that can be loaded and manipulated on a desktop computer. In *meffil*, we address this challenge by providing functions that nearly completely automate the entire process but can be replaced with calls to sets of functions that allow more detailed interaction with data processing. After each main processing step (quality control, normalization and EWAS), HTML reports are generated that summarize the results of each (Supplementary Figs S1–S3), allowing the user to evaluate the success of each step before proceeding to the next and to share with collaborators. We also provide extensively tested quality control protocols on the meffil wiki website (https://github.com/perishky/meffil/wiki). We note that EWAS in *meffil* actually fits four different regression models: no covariates and user-supplied covariates with or without surrogate variables obtained by applying SVA or ISVA.

#### *3.1.3* Analysis of mixed 450k and EPIC datasets

Given the large number of datasets that have 450k DNA methylation profiles and the apparent popularity of the new EPIC microarray, it will likely be necessary to merge 450k and EPIC datasets for analysis. This is made possible in *meffil* by applying identical methods to probes common to both microarrays. We have yet to assess the performance of this approach due to the lack of an available mixed dataset. Fortin *et al.*, 2017 have made a first attempt using the *minfi* package but their assessment dataset includes only three EPIC microarrays supplied by the manufacturer.

### 3.2 Extending FN to reduce technical variation

To assess the performance of new features in *meffil* we processed raw data from ARIES (Supplementary Methods). Although the utmost care was taken in the generation of the high-quality methylation profiles in ARIES, practical constraints lead to inconsistencies in the way samples were collected and processed. For example, DNA was extracted from a variety different sample types: whole blood, white cells, peripheral blood lymphocytes and blood spots, each with slight differences in the resulting methylation measurements. We exploit this heterogeneity to evaluate the performance of FN. An EWAS of pre-natal tobacco exposure was then applied to the cord blood samples comprising white cells and bloodspots (*n* = 777). To ensure that our observations weren’t specific to ARIES given the differences between sample starting material (e.g. blood spots versus white blood cells), we repeated the analysis in the GOYA study (Paternoster *et al.*, 2011; Supplementary Methods). Performance was assessed by comparing resulting association statistics in the two datasets to 5801 associations of a large EWAS meta-analysis of pre-natal smoking (Joubert *et al.*, 2016). As there are multiple options for selecting covariates to include in the EWAS regression model, we considered three options: no covariates, cellular composition estimates from cord or adult blood panels plus other covariates and surrogate variables obtained using ISVA (Leek and Storey, 2007; Teschendorff *et al.*, 2011). Consistent with previous findings, ISVA surrogate appear to sufficiently account for most confounding factors including heterogeneity of cellular composition (McGregor *et al.*, 2016) resulting in highly sensitive and specific EWAS findings (Supplementary Fig. S4).

#### *3.2.1* Extending FN to include fixed and random effects

We and others (Akulenko *et al.*, 2016) have found that FN often fails to completely remove slide and plate effects (Supplementary Table S1). Slide effects occur because groups of samples are measured using the same glass slide or bead chip (12 samples per slide for 450 K microarrays and 8 per slide for EPIC microarrays). Plate effects occur because groups of samples are bisulfite converted on the same 96-well plate. We therefore revised our implementation of FN to allow additional fixed and random effects to be included with the control probe summaries.

**Fig. 2.**
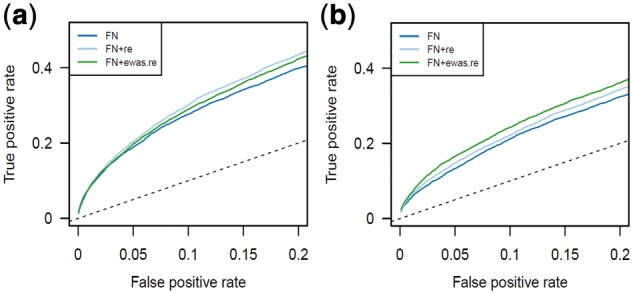
Effect of adjusting ‘slide’ or ‘plate’ as a random effect. True positive rates (TPRs) are consistently higher in a downstream EWAS when variation due to ‘slide’ effects in ARIES (**a**) and ‘plate’ effects in GOYA (**b**) are removed using random effects models. Random effects models were applied either probe quantiles along with control variation in FN (‘FN+re’) or during the EWAS (‘FN+ewas.re’). TPRs were estimated by comparison to associations from a large meta-analysis (Joubert *et al.*, 2016)

Normalization reports for ARIES showed a large drop in batch-associated variation after including batch (slide) as a random effect in FN. It was not possible to model batch as a fixed effect because induced group sizes were too small. When we performed an EWAS of pre-natal tobacco exposure in the resulting normalized version of ARIES, we observed increased specificity and sensitivity to detect previously meta-analyzed associations (Joubert *et al.*, 2016; Fig. 2). Area under the curve (AUC) increased in ARIES (0.63 to 0.65, *P* < 2.2 x 10^−16^, DeLong’s test) and in GOYA (when including plate as a random effect; 0.58 to 0.59, *P* = 1.8 x 10^−7^). For comparison, we also applied a random effects EWAS to FN normalized data. Including slide as a random effect in an EWAS of ARIES was not an improvement over our extension of FN (AUC decreased from 0.65 to 0.64, *P* = 1.8 x 10^−6^), however including plate as a random effect in an EWAS of GOYA was an improvement (AUC increased from 0.59 to 0.61, *P* < 2.2 x 10^−16^). All receiver operating characteristic (ROC) curves are shown in Figure 2. Users could use a similar approach using the normalization report and well-established EWAS findings to make normalization decisions.

#### *3.2.2* Automated parameter selection

FN has one main parameter that can be set by the user: the number of principal components (maximum = 42) derived from control features to be used to normalize the probe quantiles (Fortin *et al.*, 2014b). The default number advised by Fortin *et al.*, 2014b is two, derived as the number maximizing discovery of differentially methylated signals in a few examples. In meffil, we implemented an approach that estimates the number of principal components as the number that best explains variation in the probe intensity quantiles. This test is performed under cross validation in order to avoid overfitting (Supplementary Material).

**Fig. 3.**
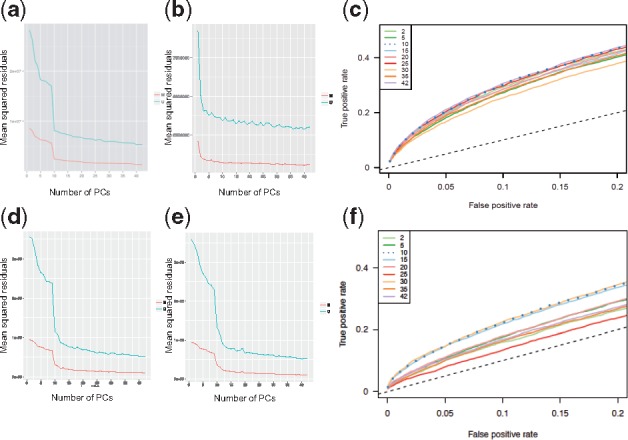
Parameter selection for FN. The main parameter for FN is the number of principal components of control variation with which to normalize probe quantiles. Screeplots (a, b, d, e) show the metric used to *meffil* for choosing the optimal number of principal components in ARIES (a, b, c) and GOYA (d, e, f), the amount of probe quantile variation unexplained by the principal components under 10-fold cross validation. The explained variation is mainly due to technical variance as the control probes should not be correlated with biological signal (Supplementary Material). Screeplots (a, d) show the variation without regressing out random effects whereas plots (b) and (e) show the variation after regressing out slide (b) or plate (e) as random effect. Plots (c) and (f) compares true and false positive rates in a downstream EWAS of pre-natal smoking in ARIES (c) and GOYA (f) after normalizing with different numbers of principal components and regressing out slide or plate as a random effect. TPRs were estimated by comparison to associations from a large meta-analysis (Joubert *et al.*, 2016)

To evaluate the performance of the automatic parameter selection, we generated nine normalizations of ARIES and GOYA cord blood samples, each normalized with a different number of control summary principal components and evaluated the sensitivity and specificity of identifying associations with pre-natal tobacco exposure. ROC curves show that parameter choice can have a large influence (Fig. 3a and b), with the recommended choice of 10 returning the best performance.

#### *3.2.3* Reducing heterogeneity in Meta-analyses with minimal data sharing

**Fig. 4.**
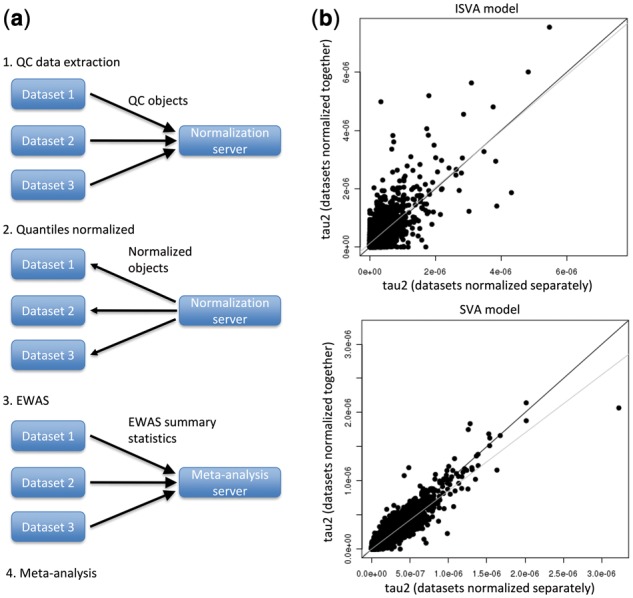
Meta-analysis with normalized data. Data can be normalized using *meffil* as illustrated in (a) by generating QC objects for each dataset, sending them to a normalization server for normalization and then sending them back to each dataset to complete normalization of each sample. (b) The heterogeneity *tau*^2^ statistic is shown for CpG sites in the meta-analyses of age performed with and without normalizing the seven datasets together prior to meta-analysis. The top plot shows heterogeneity when ISVA is used to generate surrogate covariates and the bottom plot when SVA is used instead. CpG sites shown in the plot are those identified as associated with age in the EWAS of the combined dataset, 2486 associations for ISVA and 7697 for SVA. The dark diagonal line shows *y* = *x* and the grey line the regression line

Due to the way that FN is re-implemented in *meffil*, it is possible to normalize datasets residing on distinct servers together while sharing only the control probe summaries and probe intensity quantiles between the two servers (Fig. 4a). We evaluated the effect of this approach on heterogeneity in a meta-analysis of age in seven publicly available DNA methylation datasets (*n* = 2967, Supplementary Table S2, Supplementary Methods). In a first meta-analysis, each dataset was normalized separately and, in a second meta-analysis (the ‘mega’ EWAS), the datasets were normalized together (Fig. 4a). Heterogeneity was compared between meta-analyses by paired *t*-test of *tau^2^* (Rucker *et al.*, 2008) to only those CpG sites associated with age in an EWAS of all datasets merged together (Bonferroni adjusted *P* < 0.05 and at least 0.1% change in methylation per year). In each EWAS surrogate variables generated either by ISVA or SVA were used as covariates. The ISVA mega EWAS identified 2487 CpG sites associated with age (Bonferroni adjusted *P* < 0.05 and absolute value of the regression coefficient greater than 0.1% per year) whereas the SVA mega EWAS identified 7697 sharing 1773 associations. The regression coefficients of the pooled set of 8411 associations were highly correlated between them (*R* = 0.91).

Using ISVA, heterogeneity was actually lower when datasets were normalized separately prior to meta-analysis (mean *tau^2^* difference = 1.2 x 10^−7^, *P* < 7.7 x 10^−54^; Fig. 4b). Conversely, when SVA was used, heterogeneity was much lower when datasets were normalized together (mean *tau*^2^ difference = 2.4 x 10^−8^; *P* < 1.4 x 10^−194^; Fig. 4b).

Agreement between meta-analyzed and mega EWAS was also highly covariate-dependent and followed expectations that reduced heterogeneity led to greater agreement. Agreement was quantified by treating the mega EWAS associations as the true set of associations and calculating the false discovery rate (FDR) and TPR of the corresponding meta-analysis. For ISVA-based EWAS, FDR was 35% and TPR was 63% when datasets were normalized separately. When datasets were normalized together, FDR was slightly higher (36%) and TPR lower (49%). For SVA, FDR was much lower at 6% and TPR much higher at 78% when datasets were normalized separately. When datasets were normalized together, FDR was slightly lower (4%) and TPR slightly higher (81%). From these results, we conclude that normalization prior to meta-analysis may improve results but this is not guaranteed.

### 3.3 Perfect confounding between batch effects and biological phenotypes is not resolved by functional normalization

A common problem in epidemiological datasets is perfect confounding with batch, particularly for opportunistic case-control studies in which data is generated for cases subsequent to data collected from a control population. We evaluated the efficacy of FN to remove only technical variation based on control variation while leaving biological variation intact. To test this, we compared methylation differences between methylation profiles obtained from cord blood against peripheral blood collected in adolescence under two scenarios, one in which there was perfect confounding with batch (e.g. GOYA cord against ARIES adolescence) and another in which batch was randomized (e.g. ARIES cord versus ARIES adolescence). In the unconfounded analysis, only 14 DNA methylation differences were identified (Bonferroni adjusted *P* < 0.05) after adjusting for heterogeneity in cellular composition. In contrast, in the confounded analysis, there were 38 950 methylation differences and this included only seven of the 14 differences from the unconfounded analysis. Of the 38 950, 62% had effect sizes in the same direction as in the unconfounded analysis. This suggests that the vast majority of the 38 950 were false positives.

We then asked if adjusting for controls directly in the EWAS regression model would reduce the number of apparent false positives while retaining some of the true positives. Under this model we obtained 199 differentially methylated CpG sites, of which 50 overlapped with the 38 950 from the confounded analysis and none with the unconfounded analysis. Of the 199 123 (62%) agreed on the direction of association. Once again, these results suggest that most or all of the 199 were false positives. This was not due to the control probes failing to fully account for batch variation as a few of the ‘hybridization’ controls perfectly differentiated between batches. The false positives were then possibly due to model instability due to high correlation between controls and the variable of interest.

## 4 Discussion

Illumina Infinium DNA methylation microarrays have been used in a number of large-scale epigenetic epidemiological studies due to their low cost and large coverage of the genome. Despite the extensive use of these arrays, memory efficient and comprehensive software are currently lacking. We have designed *meffil* to perform pre-processing, quality control, data harmonization, normalization and EWAS easily, flexibly and memory-efficiently. We have demonstrated that *meffil* can remove unwanted variation both using FN and by including covariates in EWAS models. Automatic generation of comprehensive reports at each step allows users to assess the success of each and potentially repeat steps after tweaking parameters to improve performance. The possibility of normalizing remote datasets together without sharing sensitive information may help to reduce heterogeneity in meta-analysis.

To evaluate different settings in *meffil*, we used the ARIES and GOYA datasets and compared associations with pre-natal tobacco exposure under various normalization schemes against those published for a large meta-analysis (Joubert *et al.*, 2016) as an example. A limitation of this approach is that the meta-analyzed set of associations might be contaminated with false positives due to batch and confounding effects that replicate across meta-analyzed datasets. Although the meta-analysis appears to be well-powered and therefore able to identify associations with small effect sizes, there are undoubtedly false negatives due to the variety of different data generation, quality control and normalization procedures applied to meta-analyzed datasets. Furthermore, all studies relied on self-reported smoking during pregnancy.

We used pre-natal smoking where multiple loci with small effect sizes contribute to the phenotypic variance rather than large case control effects (such as cancer). As batch effects will have the largest impact on such small effects, correcting for these effects in the most optimal way will improve power. In addition, integration and harmonization across different studies will lead to increased power in EWAS. However, simulations with different sizes of batch, confounder and case control effects are required to find out which method and settings work best but are not the scope of this paper. Especially, as for most traits the genomic architecture is unknown, different assumptions should be made for different traits.

We and others (Akulenko *et al.*, 2016) have noted that FN may fail to completely remove certain technical effects, either because that variation is missing from microarray controls or because probe quantiles rather than probe intensities are directly adjusted. To address the former possibility, we allow the user to include additional technical variables as fixed or random effects. As shown in Supplementary Table S1, the addition of a random ‘slide’ effect does indeed reduce variation associated with ‘slide’. For this reason, it might be better in some cases to employ a different normalization method. Crucially, we demonstrate that though FN attempts to separate technical from biological variation, when batch and phenotype are perfectly confounded results can be extremely unreliable. We recommend that cases and controls be assayed jointly within a single experiment in a random order.

Reducing heterogeneity in meta-analysis is likely to increase power to observe associations. Although we hypothesized that normalizing between datasets prior to meta-analysis could reduce heterogeneity, our analyses show that this cannot always be assumed and may depend on the regression models used for EWAS, at least for FN. Further work is needed to better understand the conditions necessary for reducing heterogeneity by normalization.

We plan in future to provide alternative background subtraction and normalization approaches, re-implemented in order to preserve the current low memory requirements of *meffil* and ability to normalize datasets present on distinct servers. We note however that for some methods, the re-implementation will not produce identical results because they depend on the entire dataset being loaded into memory [e.g. (Lehne *et al.*, 2015)]. For specific parts of the normalization pipeline, we plan to offer means for users apply their own custom R code. Future directions also include the possibility of integrating *meffil* within systems like DataShield (Gaye *et al.*, 2014) that will allow not only combined normalization but also EWAS of datasets present on distinct servers. This will improve both the power of and the speed at which meta-analyses of multiple cohort studies can be completed.

## Supplementary Material

Supplementary Methods
