# Supplementary material for: Meffil: efficient normalization and analysis of very large DNA methylation datasets
Source: Bioinformatics. 2018 Jun 21;34(23):3983–9. doi: 10.1093/bioinformatics/bty476 (PMC6247925; doi:10.1093/bioinformatics/bty476)
Supplement: Supplementary Methods [file bty476_supplemental_methods.docx]

**SUPPLEMENTAL METHODS**

**Data**

**Accessible Resource for Integrative Epigenomic Studies (ARIES)**

Samples were drawn from the Avon Longitudinal Study of Parents and Children (ALSPAC) (Boyd, et al., 2013; Fraser, et al., 2013). Blood from 1022 mother-child pairs (children at three time points and their mothers at two time points) were selected for analysis as part of Accessible Resource for Integrative Epigenomic Studies (ARIES, <http://www.ariesepigenomics.org.uk/>) (Relton, et al., 2015). Written informed consent has been obtained for all ALSPAC participants. Ethical approval for the study was obtained from the ALSPAC Ethics and Law Committee and the Local Research Ethics Committees.

Following DNA extraction, samples were bisulfite converted using the Zymo EZ DNA Methylation^TM^ kit (Zymo, Irvine, CA). Following conversion genome-wide methylation was measured using 450k arrays. The arrays were scanned using an Illumina iScan, with initial quality review using GenomeStudio. During the data generation process a wide range of batch variables were recorded in a purpose-built laboratory information management system (LIMS). The LIMS also reported quality control metrics from the standard control probes on the 450k array for each sample. Samples failing quality control were excluded from further analysis and the assay repeated. In total, there are 5469 samples for five timepoints (birth=1127; childhood=1086; adolescence=1073; pregnancy=1100; middle aged mums=1083) measured belonging to the 1022 mother-child pairs. Sample quality control and normalization was completed using with *meffil* in R version 3.2.0. Briefly, 4904/5469 ARIES samples have been successfully genotyped (Gaunt, et al., 2016). 112/5469 samples failed genotype quality control due to sample swaps, gender mismatches, high identity by descent or relatedness issues between mums and kids and were removed from ARIES. We found 411 genotype mismatches (with a concordance below 80%) between the 65 SNP probes on the 450k array and the genotype arrays with a concordance below 80% and these samples are removed. Furthermore, samples were removed if: i) mum’s samples had more than 90% concordance with a kid’s sample (22 samples) ii) concordance was below 80% between duplicates and less than 80% concordance with at least one other mums of kid’s sample (N=200). iii) mum’s samples of which concordance was below 80% between duplicates (N=10) iv) samples with low concordance (below 80%) with other timepoints (N=10). v) mum’s samples with low concordance (below 80%) with other timepoints (N=24). Methylation quality was checked by: sex check (N=191), the median intensity methylated vs unmethylated signal for all control probes (N=63), dyebias (N=14), detection pvalue (N=166), low bead numbers (N=2) and post normalization checks (N=13). Finally, 4593 samples passed quality control. Samples were normalized using FN using *meffil* (see below).

ARIES was normalized using 10 control probe principal components derived from the technical probes informed by *meffil* scree plots (Figure 3a). Principal components analysis of the normalized data using the 20,000 most variable probes shows that slide and sample type effects in cord blood were not fully eliminated by normalization (Table S1). Further investigation of these batch effects showed that slide/plate effects were confounded by sample type in this data set because, for example, different sample types were not randomized across slides and plates.

### Genetics of Overweight Young Adults (GOYA)

The Genetics of Overweight Young Adults study (GOYA) study is described in (Paternoster, et al., 2011). It includes a subset of 91,387 pregnant women recruited to the Danish National Birth Cohort (DNBC) during 1996–2002. Of 67,853 women who had given birth to a live born infant, had provided a blood sample during pregnancy and had body mass index (BMI) information available, 3.6% of these women with the largest residuals from the regression of BMI on age and parity (all entered as continuous variables) were selected for GOYA. The BMI for these 2451 women ranged from 32.6 to 64.4. From the remaining cohort, a random sample of similar size (2450) was also selected. In total, 3908 mothers were successfully genotyped. DNA methylation data were generated for the offspring of 1000 mothers in the GOYA study, equally distributed between “cases” with a BMI>32 and “controls” who were sampled from the remaining BMI distribution. All women in the GOYA study provided written informed consent that their data and biological material could be used in scientific studies of health in women and children when entering the DNBC. The GOYA study was approved by the regional scientific ethics committee and by the Danish Data Protection Board.

All data was imported into R version 3.2.0 and processed using *meffil*. In total, there are 1010 samples belonging to 1000 children. Samples were extracted from cord blood. Ten samples were poor quality samples and were therefore repeated in the lab. 933/1010 GOYA samples have been successfully genotyped. Samples were removed due to genotype mismatches between 65 genotypes extracted from the genotype and 65 SNP probes extracted from methylation arrays. Furthermore, methylation quality was checked by: sex mismatches (23 samples), the median intensity methylated vs unmethylated signal for all control probes (N=8), bisulfate 1 probes (N=8), bisulfate II probes (N=2), dyebias (N=0), detection pvalue (N=7), low bead numbers (N=0) and post normalization checks (N=5). The data was normalized using FN (Fortin, et al., 2014) and 10 principal components were used to capture technical variation. After removing low quality sample samples, we had 957 samples including 4 replicates. GOYA was normalized using 10 control probe principal components based on *meffil* scree plots. In our analyses, we included 533 samples with a normal BMI distribution.

### Publicly available data for age meta-analysis

To evaluate the performance of *meffil* in the context of meta-analyses, we obtained all datasets publicly available on the Gene Expression Omnibus (<https://www.ncbi.nlm.nih.gov/geo>) providing DNA methylation profiles of peripheral blood measured using 450k arrays made available as raw IDAT files and the age when the blood was collected. Raw IDAT files were required because FN depends on control probe intensities and these are rarely included in other data formats. We identified nine such datasets (Table S2).

## **EWAS of prenatal tobacco exposure in ARIES and GOYA**

Before analysis, samples were removed if they were replicates or due to population stratification in the genotype data. We then used three iterations to remove methylation values that were 10 SD from the mean. Associations of maternal tobacco exposure were tested in cord blood DNA methylation. To compare ‘ISVA’ to ‘all covariates’ models, we fitted maternal age, parity, maternal education, cellular composition and plate as covariates in ARIES. In GOYA, we included maternal age, parity, maternal education, cellular composition (derived from either adult or cord blood panels), plate and slide row. In all other EWAS, surrogate variables obtained by applying ISVA were included as covariates (Teschendorff, et al., 2011).

## **Automated parameter selection**

The main parameter for FN is the number of principal components of control variation with which to normalize probe quantiles. The optimal number is the smallest number that explains the most probe signal variation. In a well-designed experiment, this variance will not be correlated with biological variation and therefore represent purely technical variation.

To discover the optimal number, estimates are obtained of the probe signal variation explained by differing numbers of control probe principal components. Cross validation is used to obtain these estimates in order to avoid over-fitting and therefore removing biological variation. For each cross-validation partition of the dataset into training and testing sets, principal components are derived from the training set control probes. Different numbers of principal components together with control probe intensities in the testing set are then used to predict control probe principal components in the testing set. These predicted components are used to normalize the test set probe intensity quantiles as in FN. When cross-validation is complete, each sample has both raw quantiles and a set of normalized quantiles. The difference between these sets of quantiles represents the variance explained by the control probe principal components. The remaining variance, or residual variance, is shown in the plots.

## **Meta-analysis**

Meta analyses of age were conducted by first running an EWAS in each dataset and then meta-analyzing the summary statistics using an inverse variance fixed effects model using the *metafor* R package (Viechtbauer, 2010). The *tau^2^* metric was used to measure heterogeneity. Each EWAS was strongly concordant with published sets of age associations. The 2487 ISVA EWAS CpG sites included 346 of the 828 (Zaghlool, et al., 2015), 10 of 88 (Bocklandt, et al., 2011), 93 of 162 (Florath, et al., 2014), and 150 of 1202 (Kananen, et al., 2016) age-associated CpG sites. Similarly, the 7697 SVA EWAS CpG sites included 571 of the 828 (Zaghlool, et al., 2015), 36 of 88 (Bocklandt, et al., 2011), 122 of 162 (Florath, et al., 2014), and 337 of 1202 (Kananen, et al., 2016) age-associated CpG sites.

## **Performance evaluation**

To evaluate the performance of normalization parameters and statistical methods, an appropriate gold standard for analysis output is required. Ideally, the gold standard would be a set of EWAS associations discovered in a well powered dataset and replicated in independent data. A close approximation is the 6,073 associations of prenatal tobacco exposure in cord blood DNA methylation discovered in a recent meta-analysis of 13 birth cohorts (Joubert, et al., 2016), one of the largest EWAS studies to date. It is not, however, a perfect standard (which may not exist) due to different normalization and analysis methods used for each dataset and lack of replication testing. To ensure that the associations were not contaminated with false positives due to genetic and technical artefacts, we retained the 5,801 of the 6,073 not linked to probes potentially affected by genetic variants (minor allele frequency > 0.01 in the European subset of 1000 Genomes) or prone to non-specific binding (Naeem, et al., 2014).

We evaluated performance by constructing receiver operating characteristic (ROC) curves using the truth data described above. We used the *ROCR* package (Sing, et al., 2005) and code provided by (Maksimovic, et al., 2015) to construct the ROC curves. Since surrogate variable analysis depends on pseudo-random number generation, the EWAS function in *meffil* provides a parameter for setting the random seed prior to executing ISVA or SVA.

**References**

Bocklandt, S.*, et al.* Epigenetic predictor of age. *PloS one* 2011;6(6):e14821.

Boyd, A.*, et al.* Cohort Profile: the 'children of the 90s'--the index offspring of the Avon Longitudinal Study of Parents and Children. *International journal of epidemiology* 2013;42(1):111-127.

Florath, I.*, et al.* Cross-sectional and longitudinal changes in DNA methylation with age: an epigenome-wide analysis revealing over 60 novel age-associated CpG sites. *Hum Mol Genet* 2014;23(5):1186-1201.

Fortin, J.P.*, et al.* Functional normalization of 450k methylation array data improves replication in large cancer studies. *Genome biology* 2014;15(12):503.

Fraser, A.*, et al.* Cohort Profile: the Avon Longitudinal Study of Parents and Children: ALSPAC mothers cohort. *International journal of epidemiology* 2013;42(1):97-110.

Gaunt, T.R.*, et al.* Systematic identification of genetic influences on methylation across the human life course. *Genome biology* 2016;17:61.

Houseman, E.A.*, et al.* DNA methylation arrays as surrogate measures of cell mixture distribution. *BMC bioinformatics* 2012;13:86.

Joubert, B.R.*, et al.* DNA Methylation in Newborns and Maternal Smoking in Pregnancy: Genome-wide Consortium Meta-analysis. *American journal of human genetics* 2016;98(4):680-696.

Kananen, L.*, et al.* Aging-associated DNA methylation changes in middle-aged individuals: the Young Finns study. *BMC genomics* 2016;17:103.

Maksimovic, J.*, et al.* Removing unwanted variation in a differential methylation analysis of Illumina HumanMethylation450 array data. *Nucleic acids research* 2015;43(16):e106.

Naeem, H.*, et al.* Reducing the risk of false discovery enabling identification of biologically significant genome-wide methylation status using the HumanMethylation450 array. *BMC genomics* 2014;15:51.

Paternoster, L.*, et al.* Genome-wide population-based association study of extremely overweight young adults--the GOYA study. *PloS one* 2011;6(9):e24303.

Relton, C.L.*, et al.* Data Resource Profile: Accessible Resource for Integrated Epigenomic Studies (ARIES). *International journal of epidemiology* 2015;44(4):1181-1190.

Sing, T.*, et al.* ROCR: visualizing classifier performance in R. *Bioinformatics (Oxford, England)* 2005;21(20):3940-3941.

Teschendorff, A.E., Zhuang, J. and Widschwendter, M. Independent surrogate variable analysis to deconvolve confounding factors in large-scale microarray profiling studies. *Bioinformatics (Oxford, England)* 2011;27(11):1496-1505.

Viechtbauer, W. Conducting Meta-Analyses in R with the metafor Package. *Journal of Statistical Software; Vol 1, Issue 3 (2010)* 2010.

Zaghlool, S.B.*, et al.* Association of DNA methylation with age, gender, and smoking in an Arab population. *Clinical epigenetics* 2015;7:6.

**Figure S1:** *meffil* QC report

Plots generated in the QC report. Results are shown for 5469 samples from the ARIES resource. a. Gender prediction. b. Comparison of methylated versus unmethylated signal c. Genotype discordance using 65 SNP probes d. Proportion of detected probes by sample. e. Proportion of detected samples by probe. f. Methylation levels used to estimate cellular composition for each sample versus reference methylation profiles.


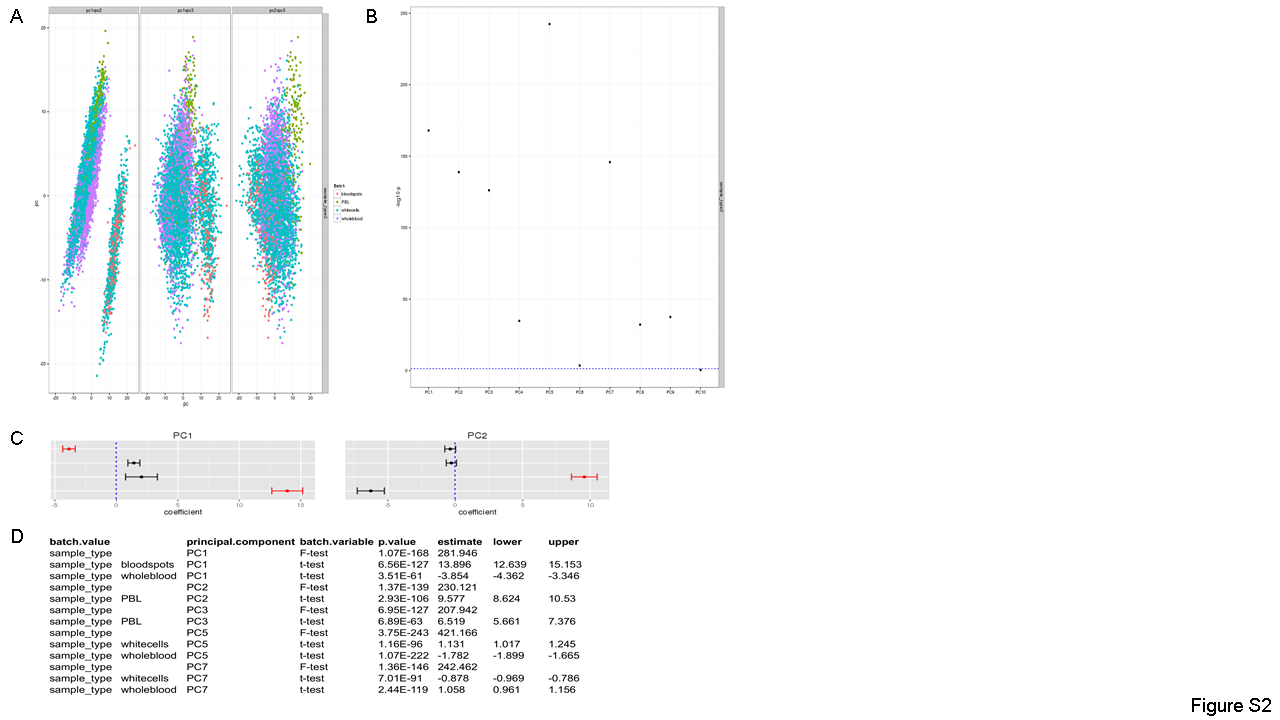


**Figure S2:** *meffil* normalization report

Plots generated in the normalization report. a. Principal component analysis plot colored by batch variable. b. ANOVA test p-values between principal components extracted from the normalized betas and batch variable. c. Coefficient plots for associations between principal components and a batch variable.

**Figure S3:** *meffil* EWAS report

Plots generated in the EWAS report. Results are shown for an EWAS on prenatal smoking in ARIES (777 cord samples). a. Manhattan plot. b. Quantile-quantile plot c. Methylation differences between cases and controls for a CpG of interest. d. Association between confounder and phenotype (variable of interest).

**Figure S4:** Performance of surrogate variables and estimates for cellular composition.

Plot compares true and false positive rates in a downstream EWAS of prenatal smoking in the ARIES dataset (a) and GOYA dataset (b). Cellular composition was estimated using the algorithm of Houseman et al. (Houseman, et al., 2012) applied to a variety of blood cell type reference panels. In addition, we show that ISVA performs well in identifying confounders in ARIES but shows a worse performance in GOYA.

**Table S1.** Association (F-statistic) between slide and data principal component in ARIES.

| **Principal component** | **Default FN** | **FN with slide random effect** |
| --- | --- | --- |
| **PC1** | 3.72 | 3.36 |
| **PC2** | 5.53 | 4.07 |
| **PC3** | 3.43 | 3.92 |
| **PC4** | 13.39 | 11.20 |
| **PC5** | 2.99 | 3.99 |

**Table S2**: Datasets used in age meta analysis

| **Dataset** | **n** | **Age mean (range)** |
| --- | --- | --- |
| GSE51032 | 516 | 53.8 (34-72) |
| GSE42861 | 689 | 51.9 (18-70) |
| GSE87571 | 732 | 47.4 (14-94) |
| GSE87648 | 384 | 36.7 (0-79) |
| GSE51057 | 329 | 53.0 (34-70) |
| GSE61496 | 312 | 48.5 (30-74) |
| GSE97362* | 235 | 13.4 (0-52) |
| GSE59065 | 97 | 52.7 (22-84) |
| GSE74548* | 87 | 70.9 (65-75) |

_* Datasets GSE74548 and GSE97362 were later removed because their EWAS of age effects were quite different from the other datasets. On average, the correlation of effects between GSE97362, and GSE74548, and those of other datasets was R=0.012 and R=0.1, respectively, whereas the average for other datasets was between R=0.19 and 0.34. The discrepancy is likely to due to their age ranges tending to be extreme compared to the other datasets._
